# Prognostic and predictive role of soluble programmed death ligand-1 in head and neck cancer

**DOI:** 10.1016/j.bjorl.2023.02.005

**Published:** 2023-02-21

**Authors:** Maria Molga-Magusiak, Anna Rzepakowska, Michał Żurek, Iwona Kotuła, Urszula Demkow, Kazimierz Niemczyk

**Affiliations:** a Medical University of Warsaw, Department of Otorhinolaryngology Head and Neck Surgery, Warsaw, Poland; b Medical University of Warsaw, Department of Laboratory Diagnostics and Clinical Immunology of Developmental Age, Warsaw, Poland

**Keywords:** Soluble biomarker, Programmed death ligand 1, Head and neck cancer, Laryngeal cancer, sPD-L1

## Abstract

**Objectives:**

The aim of the study was to investigate clinical significance of soluble PD-L1 (sPD-L1) serum level in head and neck cancer and to evaluate its role as a possible prognostic and predictive biomarker.

**Methods:**

A prospective analysis of sPD-L1 levels in 60 patients diagnosed and treated due to malignant and non-malignant lesions in the region of head and neck was performed in peripheral blood by an ELISA test.

**Results:**

The range of sPD-L1 in the study group was 0.16–1.63 ng/mL, mean 0.64 ± 0.32. There were no differences in the mean sPD-L1 regarding patients’ age, sex, and the localization of the lesion. Statistically significant difference was revealed in the average sPD-L1 level (*p* =  0.006) depending on the histopathological advancement of the lesions, 0.704 ± 0.349 and 0.512 ± 0.177 respectively in the malignant and benign group. The separate analysis of laryngeal lesions confirmed statistical difference in sPD-L1 (*p* =  0.002) for the malignant lesions (0.741 ± 0.353) compared with the benign (0.489 ± 0.175). The sPD-L1 level of 0.765 ng/mL or higher, revealed 35% sensitivity and 95.5% specificity for the diagnosis of head and neck malignant lesions (AUC = 0.664, 95% CI 0.529‒0.8, *p*-value = 0.039). The 1-year DFS was 83.3% in the group of patients with low sPD-L1 levels (< 0.765 ng/mL) and 53.8% in patients with high sPD-L1 (≥0.765 ng/mL). The 2-year OS were 68% and 69.2% respectively in both groups. The log-rank test confirmed statistically significant prognostic value of sPD-L1 level for 1-year DFS (*p*-value = 0.035).

**Conclusions:**

sPD-L1 is a promising prognostic and early recurrence predictive biomarker for head and neck cancers, most significantly for laryngeal lesions.

**Level of evidence:**

3.

## Highlights

• sPD-L1 is potential predictive biomarker for differentiation of malignant lesions in head and neck region.
• Significantly higher levels of sPD-L1 are detected in malignant tumors.
• Higher levels of sPD-L1 proves reliable prognostic biomarker in predicting early recurrence of laryngeal cancer.
• Increased sPD-L1 correlates with poor disease-free survival in head and neck cancer.

## Introduction

Head and Neck Cancers (HNCs) include malignancies of oral cavity, oropharynx, larynx, hypopharynx, salivary glands, nasopharynx and paranasal sinuses. HNCs are one of the most common malignancies worldwide, accounting for 5.3% of all cancers with 890,000 incidence, and 5.3% of cancer mortality with 507,000 deaths reported annually.^1^ Although there have been advances in diagnostic tools and novel oncological treatments were introduced, the prognosis remains poor, and outcomes barely improved over the past years. Identification of an early detection screening methods and prognostic biomarkers in HNCs is necessary to advance the accuracy of clinical investigation and risk stratification.^2^

Programmed Death protein-1 (PD-1) is an inhibitory receptor expressed on various immune response cells, including activated T-cells, B-cells, natural killer T-cells, activated monocytes and dendritic cells.^3^ Its ligand (Programmed Death Ligand-1 ‒ PD-L1) is constitutively expressed on antigen-presenting cells and is upregulated upon activation by interferons in numerous non-hematopoietic cells.^4^ PD-1/PD-L1 is a co-inhibitory immune checkpoint pathway, that has been a focal point of research in recent years. Its role is prevention of auto-aggression and maintenance of immune tolerance in peripheral tissues.

Wide range of malignant tumors are known to express PD-L1 to successively escape immune response. The main form of PD-L1 is membrane-bound PD-L1 (mPD-L1), present on the cell surface. Its soluble form (sPD-L1) can be detected circulating in the blood flow of patients with solid tumors, autoimmune diseases and some other chronic conditions.^5^

One of the causes for current attention to PD-1/PD-L1 signaling pathway are monoclonal antibodies, that target it with remarkable clinical outcomes. Pembrolizumab and Nivolumab registered in the treatment of HNCs are antibodies directed at PD-1.^6^ More anti-PD-1 (Camrelizumab, Cemiplimab, Sintilimab, Spartalizumab) as well as anti-PD-L1 (Atezolizumab, Avelumab, Durvalumab) monoclonal antibodies are indicated for treatment of cancers of different origin.^5^, ^6^, ^7^ Although the prerequisite biomarker for predicting immune checkpoint inhibitors response is tissue PD-L1 expression, association of sPD-L1 serum level with treatment outcomes is under investigation. So far, the results are contrasting: in a metastatic renal cell carcinoma higher level of sPD-L1 is associated with longer progression-free survival,^8^ but on the other hand, studies regarding a non-small cell lung carcinoma and a melanoma indicate worse treatment outcomes for patients with high expression of sPD-L1.^9^, ^10^, ^11^

In recent years a growing number of studies focused on sPD-L1 is evaluating its role as a potential biomarker. Detection of this protein is convenient, fast, repeatable and objective. So far few meta-analyses have proven that high sPD-L1 level is correlated with poor survival outcomes in numerous solid tumors.^12^, ^13^, ^14^, ^15^ To date, a significance of sPD-L1 serum level in the group of HNCs patients has not been thoroughly researched. Some promising studies concerning this group were conducted on a nasopharyngeal cancer, with the results suggestive of its role as a possible biomarker.^16^, ^17^

In this study we investigate the pretherapeutic levels of sPD-L1 in serum of patients with different sites of HNCs, dysplastic lesions and benign tumors. The data is analyzed and correlated with clinical features, histopathological staging and treatment outcomes.

## Methods

The research was approved by the local Ethics Committee, and written consent was obtained from each patient. All procedures were conducted in accordance with the ethical standards of the Declaration of Helsinki.

The prospective study included 60 patients hospitalized and treated in the Department of Otorhinolaryngology, Head and Neck Surgery between June and December 2018 due to malignant and non-malignant tumors in the region of head and neck. The inclusion criteria were age above 18 years and primary tumor diagnosis. Patients with the history of another malignancy, acute inflammatory condition, chronic inflammatory disease, systemic immunotherapy were excluded. Each participant enrolled was subjected to a surgical treatment of the lesion and was observed for minimum of two years.

The final diagnosis and staging were based on histopathological examination, conducted according to World Health Organization (WHO) classification for each type of malignancy. Laryngeal lesions were staged into adequate categories: non-dysplastic, Low-Grade Dysplasia (LGD), High-Grade Dysplasia (HGD) and invasive cancer. The study population included 17 female and 43 male participants; the mean age was 63.47 years (SD ± 14.2; median 66 years). The site of a lesion was in 46 cases larynx, in 9 cases oral cavity, in 3 cases salivary gland, and in 2 cases neck (a brachial cleft cyst). For statistical analysis the patients were divided into two groups based on a histopathological advancement. The malignant lesions group included invasive cancers (n = 34) and HGD (n = 6), and the benign lesions group consisted of non-dysplastic lesions (n = 18) and LGD (n = 2). All malignant lesions were squamous cell carcinomas Neither of the malignancy has positive expression of human papilloma virus. The non-dysplastic group clinically presented with laryngeal leukoplakia and hypertrophic mucosal changes, adenolymphoma and pleomorphic adenoma of salivary gland and two lateral neck cysts. The demography and clinical characteristics of the study population are summarized in Table 1.

**Table 1:** The demography and clinical characteristics of the study population.

	All patients	Malignant lesions	Benign lesions
Mean age (years) ± SD	63.47 ± 14.21	68.75 ± 12.05	52.9 ± 12.34
**Sex**			
Female	17	10	7
Male	43	30	13
Site			
Larynx	46	31	15
Oropharynx	9	8	1
Salivary gland	3	1	2
Neck	2	0	2

### Enzyme-linked immunosorbent assay (ELISA)

The blood samples were obtained from peripheral vein with an interval of 0–2 days before the surgical treatment. The samples were centrifuged at 4 °C to retrieve serum and further stored at −80 °C in the biological resource repository at the Department of Laboratory Diagnostics. The national standards and protocols for collecting, processing, and storing human tissues were maintained. The serum samples were thawed shortly before determination of human Programed Death Ligand-1 (PDL-1) expression by enzyme-linked immunosorbent assay (PDL-1/B7-H1/CD274 Sunred Bio ELISA kit). The ELISA detection was performed according to manufacturer’s instruction. Individual serum concentration was reported in ng/mL.

### Statistical analysis

Parameters were evaluated using IBM SPSS Statistics 26.0 and Statistica 13.3. A *p*-value lower than 0.05 was considered statistically significant for all analyses. For each statistical test, the assumptions for the tests were checked, and depending on the results, the parametric independent two-sample t-tests was used. For the graphical presentation of the results, box plots were created showing the mean, Standard Error (SE) and Standard Deviation (SD) values. Using the ROC curve analysis, the level of sPD-L1 with the highest sensitivity and specificity for differentiation between malignant and benign lesions was determined. The next part of the analysis was conducted only for malignant lesions (HGD + invasive cancer). The Kaplan-Meier curves for DFS and OS were determined depending on the critical level of sPD-L1. Log-rank test was calculated for all groups. The Cox proportional hazards model was used to quantify risk factors for recurrence and death.

## Results

### Predictive value of serum level of sPD-L1

**Figure 1:**
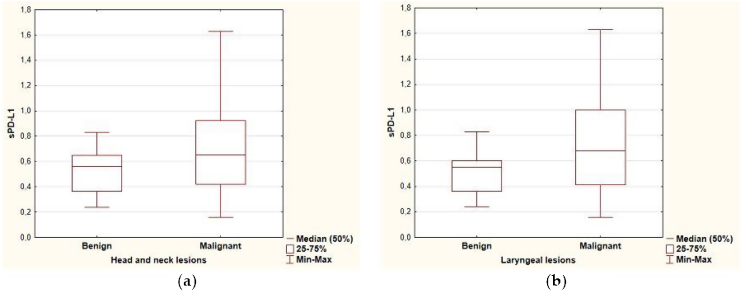
Boxplot of sPD-L1 serum level in (a) head and neck tumors, (b) laryngeal lesions.

The range of sPD-L1 serum levels in the study group was 0.16–1.63 ng/mL, with the mean value of 0.64 ± 0.32. There were no significant differences in the mean serum level of sPD-L1 according to patients’ age, sex and the localization of the lesion. The mean level of sPDL-1 was 0.704 ± 0.349 and 0.512 ± 0.177 respectively in the malignant and benign group. Based on the results of the independent two-sample *t*-test, statistical difference was revealed in the average sPD-L1 level (*p* = 0.006) depending on the histopathological staging of the lesions. The separate analysis of patients with laryngeal site also confirmed a statistical difference in the serum levels of sPD-L1 (*p* = 0.002) for the malignant group (0.741 ± 0.353) compared with the benign origin group (0.489 ± 0.175). The analysis of correlations between sPD-L1 levels and clinical staging of malignant lesions did not reveal significant differences, neither for early and advanced T-stages (Tis, T1, T2 vs. T3, T4), nor for the nodal advancement (N0 vs. N1‒N3 stage). There were no significant differences in the mean serum level of sPD-L1 in regard to histopathological tumor grading, however the data distribution was in favor of G2 (n = 20) and the grading was not provided for five patients. The comparison of sPD-L1 level with smoking status and with the history of gastroesophageal reflux disease did not reveal significant differences (Fig. 1, Table 2).

**Table 2:** The serum level of sPD-L1 in the study group regarding the histopathology and clinical data.

	Number of patients (n)	Serum sPD-L1 ng/mL (Mean, SD)	*p*-Value
**All patients**	60	0.64 ± 0.315	0.728^a^
Age ≥ 66	31	0.654 ± 0.353	
Age < 66	29	0.625 ± 0.274	
**Sex**			
Female	17	0.632 ± 0.282	0.889^b^
Male	43	0.643 ± 0.33	
**Location (all lesions)**			0.302^c^
Larynx	46	0.659 ± 0.327	
Oropharynx	9	0.529 ± 0.322	
Salivary gland	3	0.737 ± 0.05	
Neck (brachial cleft cyst)	2	0.565 ± 0.205	
**Histological types (all lesions)**			0.176^c^
Invasive cancer			
HGD	34	0.7 ± 0.364	
LGD	6	0.727 ± 0.276	
No-dysplasia	18	0.504 ± 0.166	
**Histological types (laryngeal lesions)**			**0.029**^c^
Invasive cancer	25	0.744 ± 0.374	
HGD	6	0.727 ± 0.276	
LGD	1	0.83	
No-dysplasia	14	0.465 ± 0.153	
**Histological groups (all lesions)**			**0.006**^a^
Malignant: invasive cancer + HGD	40	0.704 ± 0.349	
Benign: LGD + No-dysplasia	20	0.512 ± 0.177	
**Histological groups (laryngeal lesions)**			**0.002**^a^
Malignant: invasive cancer + HGD	31	0.741 ± 0.353	
Benign: LGD + No-dysplasia	15	0.489 ± 0.175	
**Grade (malignant lesions n = 40)**			0.635^c^
G1	3	0.627 ± 0.046	
G2	20	0.62 ± 0.347	
G3	2	0.91 ± 0.467	
**Stage – T (malignant lesions n = 40)**			0.307^b^
Tis + T1+T2	21	0.756 ± 0.32	
T3+T4	19	0.647 ± 0.38	
**Stage – N (malignant lesions n = 40)**			0.069^b^
N0	31	0.758 ± 0.354	
N1+N2+N3	9	0.518 ± 0.272	
**Stage – M (malignant lesions n = 40)**			0.124^a^
M0	39	0.723 ± 0.346	
M1	1	0.17	
**Smoking (all lesions)**			0.97^b^
No	23	0.634 ± 0.316	
Yes	37	0.644 ± 0.32	
**GERD (all lesions)**			0.429^a^
No	50	0.649 ± 0.34	
Yes	10	0.596 ± 0.143	

GERD, Gastroesophageal Reflux Disease.

^$^Analysis of variance.

a Independent two-sample *t*-test.

b Mann–Whitney *U* test.

c Kruksal–Wallis one-way analysis of variance.

**Figure 2:**
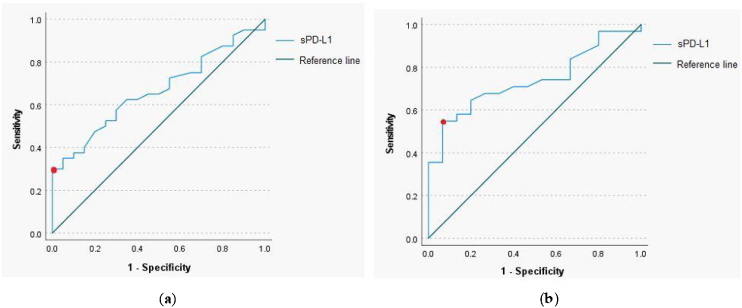
Receiver operating characteristic curve for serum sPD-L1 level to distinguish between head and neck malignant and benign lesions (a), and laryngeal malignant and benign lesions (b).

The ROC curve analysis with a calculation of the cut-off value for sPD-L1 was performed to determine predictive utility of the protein in a pretherapeutic differential diagnosis of the head and neck tumors. The analysis revealed that for the serum sPD-L1 concentration of 0.765 ng/mL or higher, the sensitivity and specificity for diagnosis of malignant lesion were respectively 35% and 95.5% (AUC = 0.664, 95% CI 0.529‒0.8, *p*-value = 0.039). The 54.8% sensitivity and 93.3% specificity for identification of malignant laryngeal lesion were established for the serum sPD-L1 concentration of 0.67 ng/mL (AUC = 0.739, 95% CI 0.597‒0.881, *p*-value = 0.009); (Fig. 2).

### Prognostic value of serum level of sPD-L1

**Figure 3:**
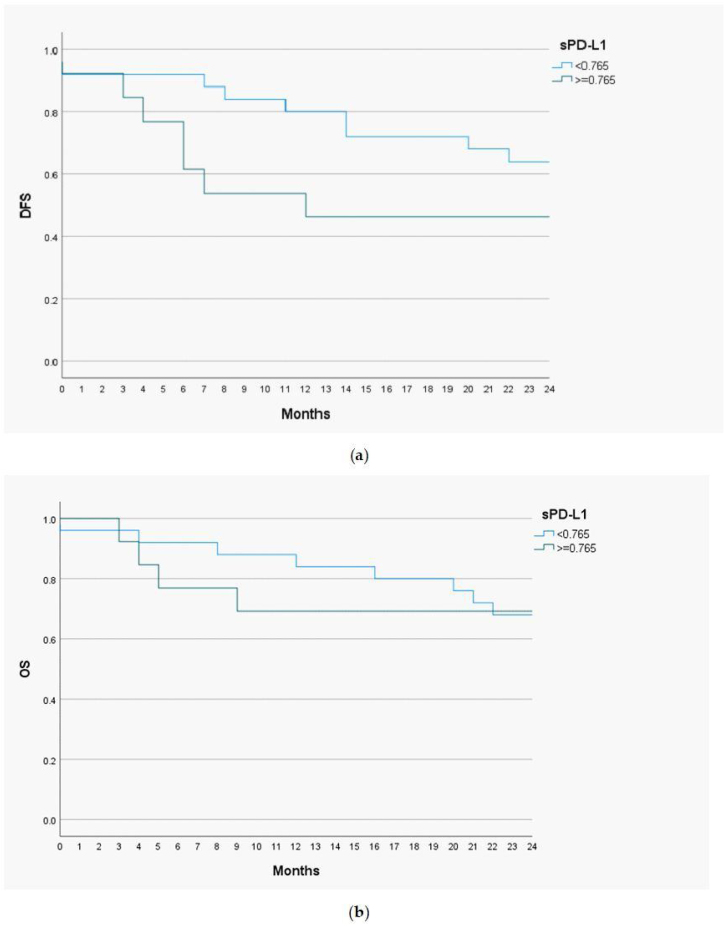
Kaplan-Meier curves representing 2-year Disease Free Survival (DFS) (a), 2-year Overall Survival (OS) (b) for sPD-L1 concentrations in the serum of patients with a head and neck cancer.

Disease Free Survival (DSF) and Overall Survival (OS) analysis with the Kaplan-Meier curves and the Cox proportional hazards models were performed to determine a prognostic utility of sPD-L1 in head and neck malignant lesions. The cut-off value of sPD-L1 was 0.765 ng/mL, as this level of biomarker had the highest predictive and prognostic value. The follow-up time for the study group was 24 months (Fig. 3).

The 1-year DFS was 83.3% in the group of patients with low sPD-L1 level (<0.765 ng/mL) and 53.8% in patients with high sPD-L1 level (≥0.765 ng/mL). The 2-year DFS in both groups were 64% and 46.2%, respectively. For the whole study population 1-year DFS amounted 73%, and 2-year DFS ‒ 57.9%. The 1-year OS valued 88% among patients with low sPD-L1 level and 69.2% among patients with high sPD-L1 level. The 2-year OS were 68% and 69.2% in both groups, respectively. The OS for study population were 81.6% for one year and 68.4% for two years. The log-rank test was calculated for all 1- and 2-years DFS and OS, but only in case of 1-year DFS the difference was statistically significant (*p*-value = 0.035).

The survival analysis was further improved by the multivariate Cox regression models for DFS and OS. The loco-regional stage (N+) was considered statistically significant and compared to the regional stage (N-), it increased the probability of recurrence on average 3.93 times (95% CI 1.28–12.1; *p*-value = 0.017) and probability of death on average 6.68 times (95% CI 1.72–21.49; *p*-value = 0.005). The remaining variables did not have a statistically significant effect on the endpoints, although it should be noted that the sPD-L1 value was at the borderline of the significance level (*p*-value = 0.062) for DFS (Table 3).

**Table 3:** The multivariate Cox regression model for serum concentration of sPD-L1 and clinical variables in head and neck cancer patients.

Variable	DFS			OS		
	HR	95% CI	*p*-Value	HR	95% CI	*p*-Value
sPD-L1 ≥ 0.765 ng/mL	2.895	0.95‒8.82	0.062	1.443	0.385‒5.41	0.586
Age	1.035	0.98‒1.09	0.184	1.057	0.99‒1.13	0.083
Male sex	1.08	0.27‒4.31	0.916	1.017	0.18‒5.61	0.985
Stage – T (Tis, T1, T2 vs. T3, T4)	1.403	0.5‒3.93	0.519	2.382	0.67‒8.42	0.178
Stage – N (N0 vs. N1, N2, N3)	3.93	1.28‒12.1	0.017	6.086	1.72‒21.49	0.005
Smokers vs. non-smokers	0.998	0.31‒3.23	0.998	0.977	0.24‒4.01	0.975

## Discussion

The ability to activate PD-1/PD-L1 immune checkpoint pathway by cancer cells is one of the most important factors contributing to successful immune escape. Binding of PD-L1 to PD-1 promotes T-cell apoptosis and exhaustion, induces inhibition of T-cell proliferation and enhances regulatory T-cells activity.^3^, ^4^ Development of such immunosuppressive microenvironment enables tumor growth and spread. PD-L1 is mainly found in membrane-bound form (mPD-L1) present on the cell surface. Recent studies highlight the presence of cancer-derived exosomes constitutively secreted from tumor cells, containing exoPD-L1.^5^, ^18^ The main focus of this study, the soluble form (sPD-L1) circulating in peripheral blood flow, is thought to be a result of proteolytic cleavage of shredded mPD-L1 and exoPD-L1. sPD-L1 can also origin from an alternative mRNA splicing.^19^ Elevated sPD-L1 level is present in the peripheral blood of patients with various types of malignancies, autoimmune or viral diseases and other conditions, including sepsis and pregnancy.^5^, ^14^, ^20^, ^21^

Pretherapeutic levels of sPD-L1 in the blood flow of cancer patients have been a focus of many studies, investigating its association with clinical features, histopathological staging, progression of a disease and treatment outcomes. Its elevated concentrations have been detected in more than twenty different types of malignancies. Higher baseline level of sPD-L1 is a predictor of worse clinical response in a peripheral T-cell lymphoma and a nasal natural killer/T-cell lymphoma.^22^, ^23^ Elevated pretherapeutic concentration is proven to correlate with worse outcomes in a hepatocellular carcinoma, a renal cell carcinoma and many other solid tumors.^24^, ^25^, ^26^ Over the course of the past few years, four meta-analyses investigating the significance of sPD-L1 level in various cancers has been conducted.^12^, ^13^, ^14^, ^15^ All of them consistently conclude that elevated sPD-L1 concentration is prognostic of worse outcomes, and most significantly is correlated with poor primary endpoint — Overall Survival (OS).

Head and neck cancers are still diagnosed in advanced stages, and despite huge impact of molecular investigations on current understanding of carcinogenesis, for the most of the HNCs we have neither prognostic nor predictive biomarkers. This study was designed to investigate whether sPD-L1 serum concentration could be used to differentiate malignant and benign lesions in the head and neck region, which has not been well researched yet. The study published by Zhang et al. in 2015 concerning Oral Squamous Cell Carcinoma (OSCC) delivered very promising results. The level of sPD-L1 was significantly related to grade, clinical stage and lymph node advancement.^27^ In 2018 Theodoraki et al. found no relevance of serum level of sPD-L1 in regard of clinicopathological features of HNCs on a cohort of 40 patients.^18^ Yang et al. investigated significance of sPD-L1 level on the group of 35 patients with Nasopharyngeal Cancer (NPC). The study revealed that a serum level of the biomarker was correlated positively with stage and nodal advancement.^16^ A year later Lu et al. confirmed importance of sPD-L1 in NPC patients by noticing correlation of sPD-L1 level combined with plasma EBV-DNA with distant metastasis-free survival.^17^ In our study we investigated the significance of pretherapeutic level of this biomarker in heterogenic group of HNCs, comparing malignant lesions (invasive cancers and HGD) to benign tumors (non-dysplastic lesions and LGD). Our analysis indicates that sPD-L1 is a potential predictive biomarker for differentiation of malignant lesions located in the head and neck region. Since most of the study population presented a laryngeal site of the tumor, this marker is particularly promising in early detection of laryngeal proliferative lesions. Diverse conclusions among studies regarding correlation of sPD-L1 levels and clinical staging require independent verification on larger study groups.

Clinical importance of proven and reliable prognostic biomarkers in oncological setting cannot be overestimated. They enable individualized, reasonable intensification or de-escalation of the therapy and the most appropriate follow-up. So far, a few meta-analyses have proven that high sPD-L1 level is correlated with poor survival outcomes in solid tumors,^12^, ^13^, ^14^, ^15^ however only the most recent one from 2021 by Huang et al. includes a HNC study.^12^ Before our report, only Lu et al. has proven sPD-L1 a negative prognostic factor for disease metastatic free survival in the head and neck region, specifically in a nasopharyngeal cancer. Therefore, our findings concerning sPD-L1 significance in predicting early (1-year) recurrence and prognosing DFS in HNCs are very valuable, even though sPD-L1 levels seemed to have no effect on overall survival.

Although our study reveals some encouraging results, it is not free of limitations. The study group was fairly small. Moreover, the distribution of cancer localization within the head and neck region was not sufficient, however the reason for that was the consecutive inclusion into the study group. The representation of patients with LGD was deficient and therefore it was not possible to assess the predictive role in regard to the histopathological staging. Another limitation of the study is the lack of comparison of serum sPD-L1 level to tissue expression of PD-L1. This analysis could be conducted retrospectively, after securing grant for further investigations. The bias regarding the prognostic role of PD-L1 may be involved with the type of curative therapy, what in the future will require analysis of large and comparable in advancement cohorts.

Presented results encourage further cohort-based and multicenter studies regarding predictive and prognostic role of serum sPD-L1 in head and neck cancers. Future research concerning comparison of sPD-L1 level with a tissue expression in this group is required for more comprehensive understanding and individualized treatment options.

## Conclusions

sPD-L1 is a promising predictive and prognostic biomarker for head and neck cancers, particularly for laryngeal lesions. Further research concerning this protein is required to fully understand its significance in HNCs.

## Authors’ contributions

Conceptualization: AR, MMM, MŻ; methodology AR, MMM, MŻ; validation, AR, MMM, MŻ, IK; investigation MMM, AR, MŻ, IK; data curation MMM, MŻ, AR; writing-original draft preparation, MMM; writing-review and editing, AR, MŻ; visualization AR, MMM, MŻ; supervision, KN, UD; project administration AR, MMM, MŻ. All authors have read and agreed to the published version of the manuscript.

## Conflicts of interest

The authors declare no conflicts of interest.
